# 2D Dynamic Directional Amplification (DDA) in Phononic Metamaterials

**DOI:** 10.3390/ma14092302

**Published:** 2021-04-29

**Authors:** Moris Kalderon, Andreas Paradeisiotis, Ioannis Antoniadis

**Affiliations:** Dynamics & Structures Laboratory, Section of Mechanical Design & Control Systems, School of Mechanical Engineering, National Technical University of Athens, 15780 Athens, Greece; aparadis@mail.ntua.gr (A.P.); antogian@central.ntua.gr (I.A.)

**Keywords:** metamaterials, phononic, dynamic directional amplifier, damping

## Abstract

Phononic structures with unit cells exhibiting Bragg scattering and local resonance present unique wave propagation properties at wavelengths well below the regime corresponding to bandgap generation based on spatial periodicity. However, both mechanisms show certain constraints in designing systems with wide bandgaps in the low-frequency range. To face the main practical challenges encountered in such cases, including heavy oscillating masses, a simple dynamic directional amplification (DDA) mechanism is proposed as the base of the phononic lattice. This amplifier is designed to present the same mass and use the same damping element as a reference two-dimensional (2D) phononic metamaterial. Thus, no increase in the structure mass or the viscous damping is needed. The proposed DDA can be realized by imposing kinematic constraints to the structure’s degrees of freedom (DoF), improving inertia and damping on the desired direction of motion. Analysis of the 2D lattice via Bloch’s theory is performed, and the corresponding dispersion relations are derived. The numerical results of an indicative case study show significant improvements and advantages over a conventional phononic structure, such as broader bandgaps and increased damping ratio. Finally, a conceptual design indicates the usage of the concept in potential applications, such as mechanical filters, sound and vibration isolators, and acoustic waveguides.

## 1. Introduction

Phononic metamaterials are manmade, periodic structures whose periodicity can be in the material phases, the internal geometry, or the boundary conditions. The main feature of elastic metamaterials is their ability to attenuate waves in specific frequency ranges, known as stopbands or bandgaps. These bandgaps can be broadly classified in two physical mechanisms: Bragg scattering [1] and local resonance [2,3,4]. In general, the Bragg-type bandgaps occur at wavelengths in the direction of sound wave propagation with the same order of magnitude as the lattice size and require large lattice constants [5]. On the other hand, locally resonant bandgaps correspond to internal resonances due to the microstructure, and they can be generated using resonators [6,7,8,9,10,11]. Both types of filtering can be achieved by customizing the structure of the unit cell [2,12,13]. For example, researchers usually obtain low-frequency Bragg gaps by embedding high-density materials in low-density host materials, by constructing large unit cells to retain low wave speed or by using large lattice constants [12,14,15,16]. On the contrary, locally resonant bandgaps may be easily obtained at low frequencies, yet, they require heavy resonators to obtain wide bandwidths, which may prohibit their practical implementation.

Recently, attempts have been made towards artificially increasing the inertia of the resonating mass via negative stiffness elements or amplification mechanisms. For example, the inclusion of negative stiffness elements to the oscillating system shows promise in addressing this issue to a certain extent, revealing the potential of the KDamper (KD) concept [17,18,19] towards the design of low-frequency acoustic meta-materials [20,21,22]. Similarly, inspired by the successful application of inertial amplifiers in engineering applications, several works proved their suitability in the context of periodic structures [23,24,25,26]. Kulkarni and Manimala [27] studied the longitudinal elastic wave propagation characteristics of inertant acoustic metamaterial configurations having inerters either in the local attachments or in the lattice, using effective models for their one-dimensional discrete element lattice chains. Their analysis showed that up or downshifting the bandgap frequency range and its extent depends on the inerter configuration while retaining static mass-addition to the host structure to a minimum. Yilmaz et al. [24] first embedded amplification mechanisms to increase the effective inertia of phononic lattices. It was observed that wide phononic bandgaps could be formed at low frequencies without using an excessive mass on the amplification mechanisms [28]. Frandsen et al. [29] investigated wave motion in a continuous elastic rod with periodically attached inertial amplification mechanisms, utilized in a manner that alters the intrinsic properties of the continuous structure. The conclusion was that the inertial amplification system is superior in terms of the added mass magnitude, as the local resonance system requires an approximately twenty times heavier mass to obtain a comparable bandgap width. Li and Li [30] extended Frandsen et al.’s model and included inertial amplification to infinite elastic beams. Other notable implementations include platonic crystals with spiral resonators [31,32], which could emerge as good alternatives due to their low-cost production merits.

In this paper, a dynamic directional amplifier (DDA) is proposed to enhance the bandgap characteristics of phononic lattices. In principle, directional amplifiers can be realized by imposing kinematic constraints at the degrees of freedom (DoFs) of the structure, increasing the inertia in the desired direction of motion. Such mechanisms have been used in wave energy conversion concepts [33]. However, there are limited publications, especially in the field of mechanical metamaterials. Devices that exploit the rotational inertia of the resonator, such as chiral structures [34,35,36,37,38] or gyroscopic spinners [29], are the most popular designs of directional amplifiers in periodic structures, where the longitudinal motion of the finite size mass elements is coupled to their rotation, following a spin. Directional amplification systems have also been implemented in other scientific fields, such as quantum information processing [39] in terms of controlling amplification and directionality of electromagnetic signals [40] as well as in photonic systems [41,42]. However, a different approach has been followed in those cases.

Here, the novelty lies in the simplicity of the proposed system. The directional amplification mechanism is realized without additional masses or complex geometries since the amplification can be achieved by coupling the kinematic DoFs of the mass with a rigid link. This simplicity is especially important for applications, including mechanical filters, sound and vibration isolators, and acoustic waveguides.

The objective of this study is to indicate the characteristics of the directional amplification-induced phononic bandgaps and provide the theoretical framework. First, the directionality effects of dampened 2D phononic lattices with coupled DoFs are illustrated, and then the analytical formulation of the phononic lattice with DDA is presented. The isolation/absorption properties of the proposed structure are exhibited from the corresponding dispersion curves, and the beneficial effect of the DDA is shown by a simple case study. Additionally, the number of unit cells is examined, showing that even finite lattices with a small number of unit cells can provide deep bandgaps with small masses. The emergent metadamping [43] phenomenon is investigated accruing from the dynamic amplification of the masses. Finally, based on these observations, an indicative conceptual design of the metastructure is demonstrated, revealing its potential for many applications.

## 2. Theory

The periodic structures to be considered comprise a finite number of identical unit cells. These unit cells are the repetitive units that are used to describe the microstructure. If the unit cells are inhomogeneous, i.e., made up of different masses and springs, the corresponding structure is periodic, whereas, with a homogeneous unit cell, the structure is also homogeneous.

### 2.1. Bloch’s Theorem

Bloch’s theorem [44] allows considering a single unit cell for studying wave propagation in the entire lattice structure. If the radius vectors of lattice points in a unit cell are denoted by x and the arbitrary displacement component of one such point by u, then assuming a harmonic plane wave solution, the displacement component is of the form:

(1) $$u_{p,q}(t)={\hat{U}}_{p,q}\;e^{st}=\tilde{U}\;e^{{ikx}_{p,q}st}$$

where $\;\tilde{U}$ is the real wave amplitude and ${k=k}_{x}{i+k}_{y}j$ represents the wave vector, $x_{p,q}{=p\alpha }_{x}{i+q\alpha }_{y}j$ defines the position of the unit cell in the lattice, and s is a complex frequency function that permits wave attenuation in time. The interatomic distances along the two in-plane directions are defined by $\alpha_{x}{,\;\alpha }_{y}$ and the spatial part of the solution can be written as:

(2) $${\hat{U}}_{p,q}=\tilde{U}\left(\mu \right)e^{{i(p\mu }_{x}{+q\mu }_{y})}$$

where ${\mu =\mu }_{x}{i+\mu }_{y}j$ is the propagation vector, whose components are $\mu_{x}{=k}_{x}\alpha_{x}$ and $\mu_{y}{=k}_{y}\alpha_{y}$.

For the case of general damping, it is not possible to set up a Bloch eigenvalue problem in the usual way because of the presence of the viscous damping term. We, therefore, resort to converting the second-order problem to a first-order problem through Laplace transformation [45]. In the absence of damping, s=±iω, so that the usual form of Bloch’s theorem is recovered. In the presence of damping, the real part of s represents the attenuation of the wave in which case:

(3) $$s\left(k\right)=-\zeta \left(k\right){\omega (k)\;\pm \;i\omega }_{n}\sqrt{1-\zeta^{2}\left(k\right)}$$

where ζ(k) is the wavenumber-dependent damping ratio and ω(k) the resonant frequency corresponding to modes. The imaginary part is the frequency of the damped wave propagation $\omega_{d}{(k)=\omega }_{n}\sqrt{1-\left(k\right)}$ with $\omega_{n}$ denoting the natural frequency of the system. Corresponding to each eigenvalue, the damping ratio is defined as:

(4) ζ(k)=−Re[s(k)]|ω(k)|

### 2.2. Simple 2D Monoatomic Lattice

**Figure 1:**
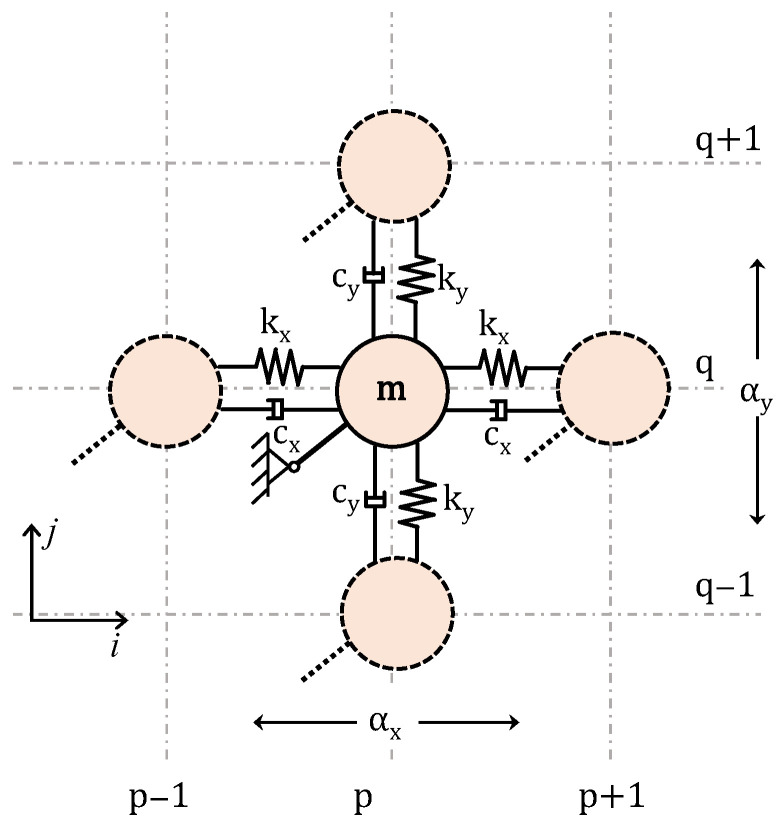
2D monoatomic lattice with detail of the unit cell.

The establishment of the theoretical framework begins considering the simple damped 2D monoatomic lattice of Figure 1, where m is the mass, $k_{x}$, $k_{y}$ the springs stiffness and $c_{x},\;c_{y}$ the viscous damping elements are connecting the masses. In general, if the model’s material is linearly elastic and geometric nonlinearity is disregarded, the deformation and interaction between the horizontal and vertical springs are assumed to have a negligible effect on the stiffness of the springs, i.e., the deformation of one spring does not affect the stiffness of either spring and the stiffness parameters of springs can be deduced separately and independent of the level of deformation.

The set of equations that describe the harmonic motion of a typical unit cell at location p, q can be expressed as:

(5) $$m{\ddot{u}}_{p,q}+k_{x}\left(u_{p,q}-{\;u}_{p-1,q}\right)+c_{x}\left({\dot{u}}_{p,q}-{\dot{u}}_{p+1,q}\right)+k_{x}\left(u_{p,q}-u_{p+1,q}\right)=0\;m{\ddot{v}}_{p,q}+k_{y}\left(v_{p,q}-v_{p,q\;-1}\right)+c_{y}\left({\dot{v}}_{p,q}-{\dot{v}}_{p+1,q}\right)+{\;k}_{y}\left(v_{p,q}-v_{p,q+1}\right)=0$$

According to Bloch’s theorem, the following wave propagation conditions are imposed to relate the displacement of the mass at location p, q with the displacements of the neighboring masses:

(6) $$u_{p,q}={\tilde{U}}_{p,q}{\;e}^{{ik}_{x}{p\alpha }_{x\;}{+ik}_{y}{q\alpha }_{y}}e^{st}={\tilde{U}}_{p,q}{\;e}^{{ip\mu }_{x}{+iq\mu }_{y}}e^{st}\;u_{p-1,q}={\tilde{U}}_{p,q}{\;e}^{{ik}_{x}(p\;-{\;1)\alpha }_{x}{+ik}_{y}{q\alpha }_{y}}e^{st}={\tilde{U}}_{p,q}{\;e}^{i(p-{1)\mu }_{x}{+iq\mu }_{y}}e^{st}=u_{p,q}e^{-{i\mu }_{x}}\;u_{p+1,q}={\tilde{U}}_{p,q}{\;e}^{{ik}_{x}{(p+1)\alpha }_{x}{+ik}_{y}{q\alpha }_{y}}e^{st}={\tilde{U}}_{p,q}e^{{i(p+1)\mu }_{x}{+iq\mu }_{y}}e^{st}=u_{p,q}e^{{i\mu }_{x}}\;v_{p,q}={\tilde{V}}_{p,q}{\;e}^{{ik}_{x}{p\alpha }_{x}{+ik}_{y}{q\alpha }_{y}}e^{st}={\tilde{V}}_{p,q}{\;e}^{{ip\mu }_{x}{+iq\mu }_{y}}e^{st}\;v_{p,q\;-1}={\tilde{V}}_{p,q}{\;e}^{{ik}_{x}{p\alpha }_{x}{+ik}_{y}(q-{1)\alpha }_{y}}e^{st}={\tilde{V}}_{p,q}{\;e}^{{ip\mu }_{x}+i(q-{1)\mu }_{y}}e^{st}=v_{p,q}e^{-{\;i\mu }_{y}}\;v_{p,q\;-1}={\tilde{V}}_{p,q}{\;e}^{{ik}_{x}{p\alpha }_{x}{+ik}_{y}{(q+1)\alpha }_{y}}e^{st}={\tilde{V}}_{p,q}{\;e}^{{ip\mu }_{x}{+i(q+1)\mu }_{y}}e^{st}=v_{p,q}e^{{i\mu }_{y}}$$

where ${\tilde{U}}_{p,q}$, ${\tilde{V}}_{p,q}$ are the wave amplitudes at nodes p, q. Then, $\alpha_{x}{,\;\alpha }_{y}$ are the unit cell dimensions and $\mu_{x}{,\;\mu }_{y}$ are the normalized wavenumbers in x, y directions.

Substitution of Equation (6) and their derivatives into Equation (5) and utilization of the trigonometric transformation $\beta =2\;-{\;(e}^{i\mu }+e^{-i\mu })=2(1\;-\;cos\mu )$, leads to the following compact matrix notation:

where

(7) $$(\;-s^{2}M+sC+K)u=0$$

(8) $$M=\left[\begin{array}{cc} m & 0\\ 0 & m \end{array}\right]\;K=\left[\begin{array}{cc} {2k}_{x}(1\;-{\;\cos(\mu }_{x}) & 0\\ 0 & {2k}_{y}(1\;-{\;\cos(\mu }_{y}) \end{array}\right]\;\;u=\left[\begin{array}{c} u_{p,q}\\ v_{p,q} \end{array}\right]$$

Assuming for simplicity that $k_{x}{=k}_{y}{=k}_{o}{=m\omega }_{0}^{2}$ and that system is undamped ($\zeta_{x}=\zeta_{y}=0$), allows rewriting Equation (7) in the following nondimensional form [3]

**Figure 2:**
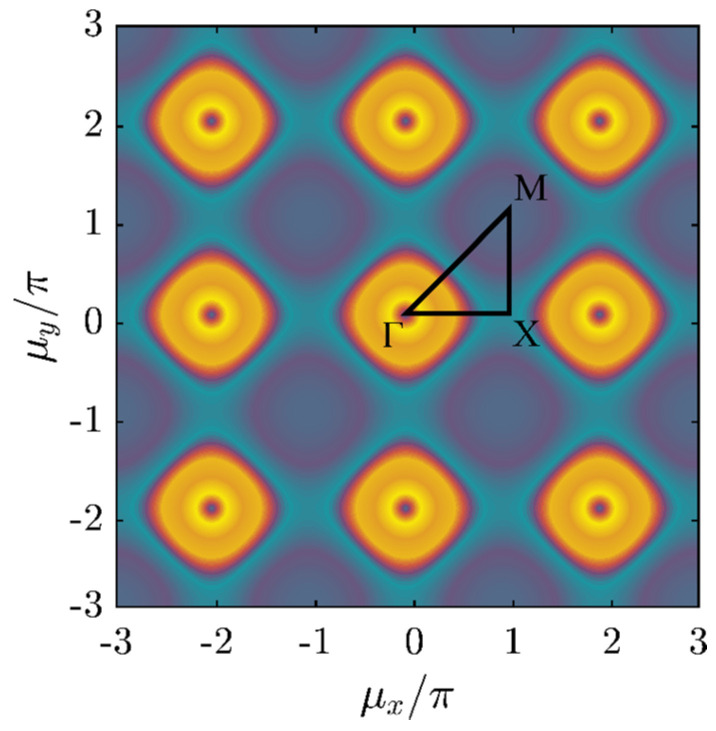
Dispersion surface of the 2D spring–mass lattice with spring stiffness, $k_{x}{,\;k}_{y}$.

The dispersion relation describes a surface in terms of the components of the propagation vector. Figure 2 shows a color map of the dispersion surface, where the third dimension is frequency ω. The plot clearly illustrates the periodicity of the surface in the wavenumber domain and highlights the “first Brillouin zone” [46] Γ-M-X-Γ and ΓΧ-ΧΜ. The fundamental period is defined by $\mu_{x}{,\;\mu }_{y}\;\in \;[\;-\pi ,+\pi ]$:

(9) $$\omega^{2}{=2\omega }_{0}^{2}\left(2-{\;\cos\mu }_{x}-{\;\cos\mu }_{y}\right)$$

### 2.3. Overview of the Dynamic Directional Amplification (DDA) Mechanism

**Figure 3:**
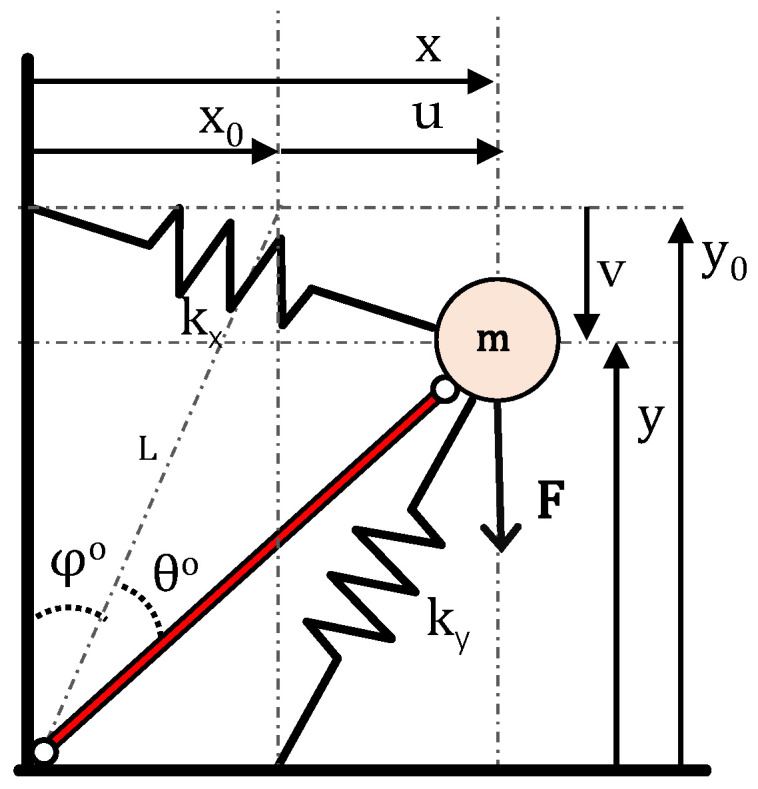
Dynamic directional amplification (DDA) mechanism, where the motion v of mass m is kinematically constrained to the motion u.

Next, the basic layout of the DDA mechanism as depicted in Figure 3 is considered. Connecting the amplification mass to the origin of the local CSYS via a hinged, rigid, massless rod imposes a kinematic constraint between the DoFs u and v. The lumped parameter model is described by the coordinates of the amplification mass (m) at a random position ${A(x,y)=(x}_{0}{+u,\;y}_{0}-v)$. The initial angle between the vertical axis and the link is denoted by $\varphi^{{}^{\circ}}=atan(\frac{x_{0}}{y_{0}})$, while the rotation of the rod by $\theta^{{}^{\circ}}$ at the random position A of the moving mass. Then the displacements of the neighboring masses (m) will cause amplification to mass m. Via the application of Lagrange’s principle and after linearization (see Appendix A), the mechanisms governing the equation of motion comes as

(10) $$m\left[{1+\tan}^{2}\left(\varphi \right)\right]\ddot{u}+\left[k_{x}{+k}_{y}{\;\tan}^{2}\left(\varphi \right)\right]u=F$$

### 2.4. 2D Monoatomic Lattice with Dynamic Directional Amplifiers (DDA)

**Figure 4:**
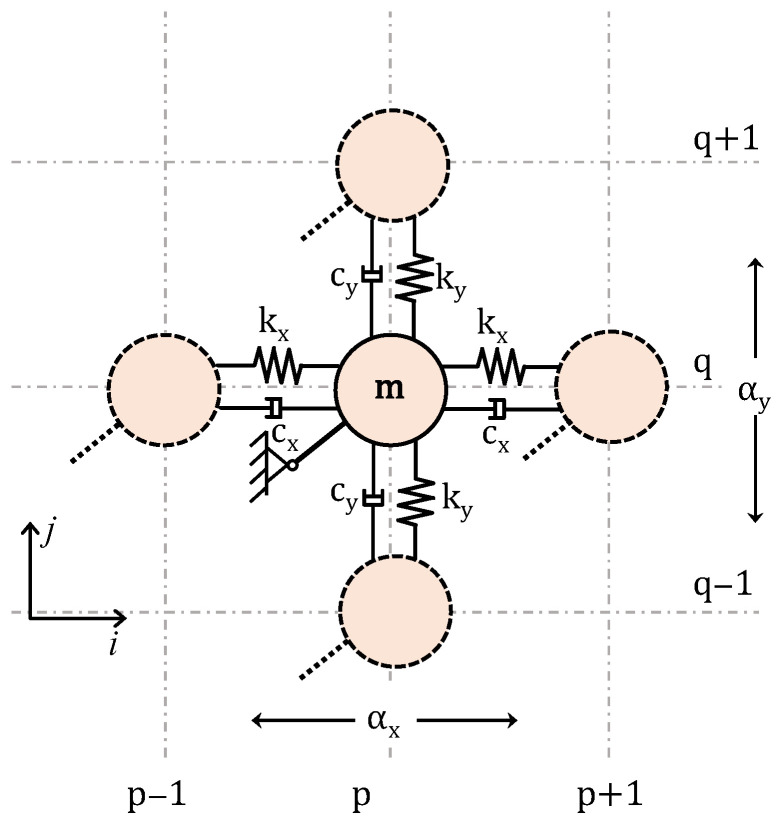
Unit lattice of a 2D periodic kinematically constrained system of masses.

Once the equation of motion of the DDA is formulated, the properties of directionality in a lattice comprising of masses connected to DDA mechanisms, as in Figure 4, can be investigated.

By setting ρ=tanϕ, the coupling of u, v DoFs is expressed as:

(11) $$u=\left[\begin{array}{c} u_{p,q}\\ v_{p,q\;} \end{array}\right]=\left[\begin{array}{c} 1\\ \rho \end{array}\right]u_{p,q}{=q\;u}_{p,q}$$

Then, by the left and right multiplication of the dynamic equation of motion with the eigenvector:

(12) $$(-s^{2}q^{T}{\mathrm{Mq}+sq}^{T}\mathrm{Cq}+q^{T}\mathrm{Kq})u=0$$

the generalized expression is obtained for the calculation of the dispersion relations.

Again, for the case of an undamped system, the existence of nontrivial harmonic plane–wave solutions requires that

(13) $$-{\;\omega }^{2}m_{a}+{2k}_{x}\left(1\;-{\cos\mu }_{x}\right)+{2k}_{ya}\left(1-{\cos\mu }_{y}\right)=0$$

where $m_{a}=m\left({1+\rho }^{2}\right){\;\mathrm{and}\;k}_{ya}{=\rho }^{2}k_{y}$.

Defining $\omega_{x}^{2}{=k}_{x}/m$ and $\omega_{y}^{2}{=k}_{y}/m$ Equation (13) is simplified to

(14) $$-{\;\omega }^{2}+{2\omega }_{x}\cos^{2}\phi \left(1-{\cos\mu }_{x}\right)+{2\omega }_{y}^{2}\sin^{2}\phi \left(1-{\cos\mu }_{y}\right)=0$$

**Figure 5:**
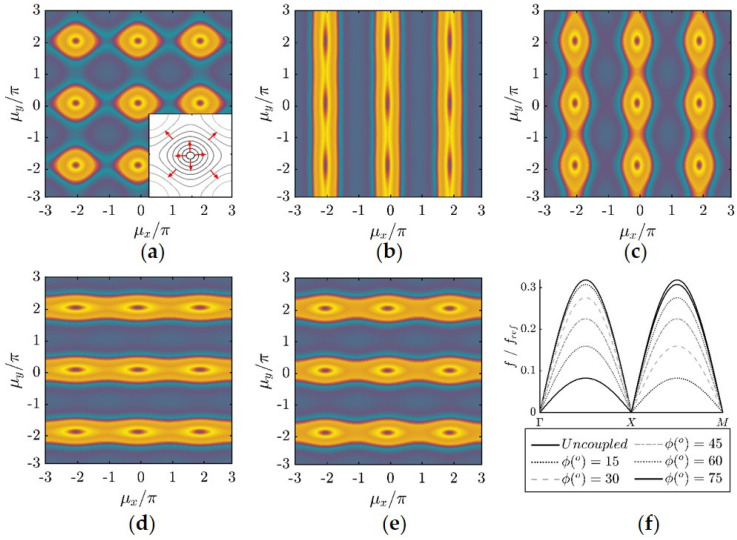
Dispersion surface of the 2D monoatomic lattice of periodic kinematically constrained DoFs with spring stiffness, $k_{x}{=k}_{y}$. (**a**) $\phi {(}^{{}^{\circ}})=15$, (**b**)$\;\phi {(}^{{}^{\circ}})=30$, (**c**)$\;\phi {(}^{{}^{\circ}})=45$ (**d**) $\phi {(}^{{}^{\circ}})=60$, (**e**) $\phi {(}^{{}^{\circ}})=75$ and (**f**) dispersion curves.

Figure 5a–e shows the contour plot of the phase constant for coupling angles $\phi {(}^{{}^{\circ}})=15$ to 75. This representation is particularly convenient as it allows visualizing the direction of the energy flow from evaluating the perpendicular directions to the iso-frequency lines. An example of the unit cell’s iso-frequency lines is provided as a key in Figure 5a. The dispersion curves are plotted in Figure 5f in the ΓΧ-ΧΜ domain against the normalized frequency ${f/f}_{\mathrm{ref}}$, where $f_{ref}={\;\omega }_{x}/2\pi$. As expected, increasing the coupling angle $\phi {(}^{{}^{\circ}})$ between the two springs reduces the maximum frequency on the dispersion curve on the ΓX plane and increases it on the dispersion curve on the XM plane. In this regard, this simple lattice configuration shows interesting characteristics. The energy flow in the case $\phi {(}^{{}^{\circ}})=15$, occurs at both planes. Further increasing this angle, wave propagation occurs mainly in one plane. The phenomenon, where wave propagation at a certain frequency is restricted to only certain directions, denotes the directionality of the periodic structure.

### 2.5. 2D Phononic Lattice with Dynamic Directional Amplifier (DDA)

#### 2.5.1. Wave Dispersion Analysis

**Figure 6:**
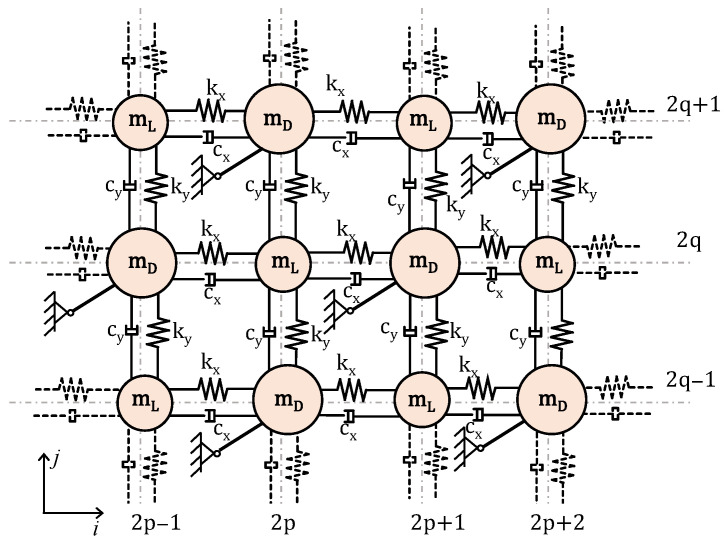
2D phononic lattice with dynamic directional amplifiers (DDA).

The infinite mass–spring dashpot lattice with the DDA of the phononic metamaterial is depicted in Figure 6. The lattice is no longer homogeneous due to the presence of two different masses $m_{L}$ and $m_{D}$. $k_{x}{,\;k}_{y}$, $m_{L}$ and $m_{D}$ form the structural backbone of the lattice, where the angle $\phi {(}^{o})$ determines the amplification angle generated by the rigid links. The components $c_{x}{,\;c}_{y}$ of the viscous damping are expressed in terms of the actual to critical damping ratios $\zeta_{i}$, from the relation $\zeta_{i}=c_{i}/(2\sqrt{m_{i}k_{i}}\;$. Considering the case of the lattice under harmonic excitation at a frequency lower than the resonance frequencies of the amplification mechanisms, then the relative motion of the structural nodes will cause amplified motion for the masses $m_{D}$ generating amplified inertial forces. Then, the wave propagation characteristics of this lattice are determined from the irreducible unit cell via Bloch’s theorem.

The equations describing the harmonic motion of a typical unit cell at a location p, q without the directional amplifying mechanism can be expressed as:

(15) $$m_{L}{\ddot{u}}_{2p,2q}+c_{x}\left({\dot{u}}_{2p,2q}-{\dot{u}}_{2p-1,2q}\right)+c_{x}\left({\dot{u}}_{2p,2q}-{\dot{u}}_{2p+1,2q}\right)+k_{x}\left(u_{2p,2q}-u_{2p-1,2q}\right)+k_{x}\left(u_{2p,2q}-u_{2p+1,2q}\right)=0\;m_{L}{\ddot{v}}_{2p,2q}+c_{y}\left({\dot{v}}_{2p,2q}-{\dot{v}}_{2p,2q-1}\right)+c_{y}\left({\dot{v}}_{2p,2q}-{\dot{v}}_{2p,2q+1}\right)+k_{y}\left(v_{2p,2q}-v_{2p,2q-1}\right)+k_{y}\left(v_{2p,2q}-v_{2p,2q+1}\right)=0\;m_{D}{\ddot{u}}_{2p+1,2q}+c_{x}\left({\dot{u}}_{2p+1,2q}-{\dot{u}}_{2p,2q}\right)+c_{x}\left({\dot{u}}_{2p+1,2q}{\dot{u}}_{2p+2,2q}\right)+k_{x}\left(u_{2p+1,2q}-u_{2p,2q}\right)+k_{x}\left(u_{2p+1,2q}-u_{2p+2,2q}\right)=0\;m_{D}{\ddot{v}}_{2p+1,2q}+c_{y}\left({\dot{v}}_{2p+1,2q}-{\dot{v}}_{2p+1,2q-1}\right)+c_{y}\left({\dot{v}}_{2p+1,2q}-{\dot{v}}_{2p+1,2q+1}\right)+k_{y}\left(v_{2p+1,2q}-v_{2p+1,2q\;-1}\right)+k_{y}\left(v_{2p+1,2q}-v_{2p+1,2q+1}\right)=0$$

For each of the system of Equation (15), assuming a plane–wave solution leads to the following relations between the DoFs of the lattice:

(16) $$u_{2p-1,2q}{=u}_{2p+1,2q}e^{-{i\mu }_{x}}\;u_{2p+2,2q}{=u}_{2p,2q}e^{{i\mu }_{x}}\;v_{2p,2q-1}{=v}_{2p+1,2q}e^{-\frac{{i\mu }_{x}}{2}}e^{-\frac{{i\mu }_{y}}{2}}\;v_{2p,2q+1}{=v}_{2p+1,2q}e^{-\frac{{i\mu }_{x}}{2}}e^{\frac{{i\mu }_{y}}{2}}\;v_{2p+1,2q-1}{=v}_{2p,2q}e^{\frac{{i\mu }_{x}}{2}}e^{-\frac{{i\mu }_{y}}{2}}\;v_{2p+1,2q+1}{=v}_{2p,2q}e^{\frac{{i\mu }_{x}}{2}}e^{\frac{{i\mu }_{y}}{2}}$$

Substituting these relations into the equations of motion and incorporating the conditions for unit cell periodicity yields four homogeneous equations, which written in matrix form, is:

(17) $$M_{p}\ddot{u}+C_{p}\dot{u}+K_{p}u=0$$

Finally, the system with the amplified masses $m_{D}$ can be calculated by multiplying $M_{p}{,\;C}_{p}$ and $K_{p}$ by the transformation matrix $Q_{p}$.

The dispersion relationship of the phononic structure with directional inertial amplifiers is given by:

(18) $$\det\left[-s^{2}M_{p,\alpha }+{sC}_{p,\alpha }+K_{p,\alpha }\right]=0$$

where

(19) $$M_{p,\alpha }{=Q}_{p}^{T}M_{p}Q_{p}\;C_{p,\alpha }{=Q}_{p}^{T}C_{p}Q_{p}\;K_{p,\alpha }{=Q}_{p}^{T}K_{p}Q_{p}$$

The band structure of the infinite lattice is obtained through solving Equation (17) for the lattice without the amplification mechanism and Equation (18) for the lattice with the amplification mechanism for s by evaluating μ. A more detailed description of the matrices is provided in Appendix B. In this work, complete bandgaps are considered, i.e., the frequency ranges in which no branch exists regardless of mode.

#### 2.5.2. Structural Dynamics of the Finite Lattice

Since the phononic lattice of Figure 6 is described by a series of discrete mechanical elements, the profile of the propagating waves is captured by the discretized displacement. Consequently, the equation of motion of the metamaterial, for $M_{x}{\times \;M}_{y}$ number of units is expressed in matrix formulation as:

(20) $$M\ddot{u}(t)+C\dot{u}{(t)+\mathrm{Ku}(t)=Fe}^{st}$$

where $M_{m\times m}$, $C_{m\times m}$, $K_{m\times m}$, ${\ddot{u}}_{m\times 1}$, ${\dot{u}}_{m\times 1}$ $u_{m\times 1}$, $F_{m\times 1}$ and m is the number of degrees of freedom (Dofs) of the metamaterial. For the lattice without the amplification mechanism, ${m=2M}_{x}M_{y}$. Similarly, for the lattice with the DDA, ${m=3/2\;M}_{x}M_{y}$ and the global mass ${[M}_{p,\alpha }^{G}]$, damping ${[C}_{p,\alpha }^{G}]$ and stiffness ${[K}_{p,\alpha }^{G}]$ matrices can be calculated as follows:

(21) $$M_{p,\alpha }^{G}{=Q}_{G}^{T}M_{p}^{G}Q_{G},\;C_{p,\alpha }^{G}{=Q}_{G}^{T}C_{p}^{G}Q_{G}\;K_{p,\alpha }^{G}{=Q}_{G}^{T}K_{p}^{G}Q_{G}$$

For $M_{x}{=M}_{y}=2$ the global mass ${[M}_{p,\alpha }^{G}]$, damping ${[C}_{p,\alpha }^{G}]$ and stiffness ${[K}_{p,\alpha }^{G}]$ matrices are given in Appendix C. For $M_{x}{,\;M}_{y}>2$ stiffness ${[K}^{G}]$, damping ${[C}^{G}]$ and mass ${[M}^{G}]$ matrices of each unit cell are assembled as in any finite element analysis to produce the Global matrices of the periodic structure. Assuming that the finite lattice is excited harmonically at certain input nodes with frequency ω, means that the force magnitude vector F is zero everywhere except in the row or column corresponding to the component of the input node for which it has unit amplitude. The transfer matrix is then calculated as:

(22) $$TF={\left(-s^{2}M+\;sC+K\right)}^{-1}F,$$

and the frequency response function (**FRF**) of the metamaterial is defined as:

(23) FRF=20log(TF)

## 3. Numerical Results

**Figure 7:**
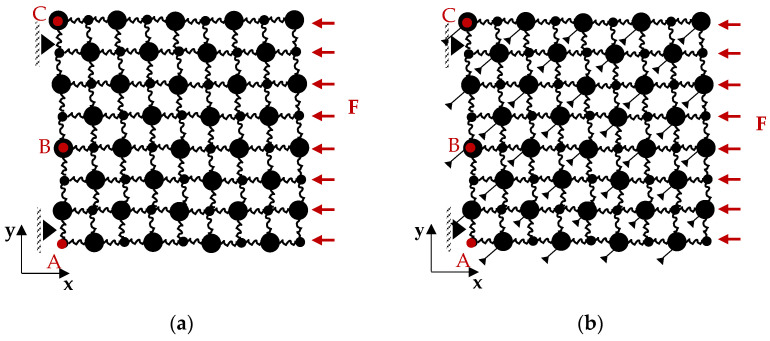
Structure with $M_{x}{\;\times \;M}_{y}$ unit cells with a periodic loading acting at the right boundary and simple supports at the left corners (**a**) without DDA (**b**) with DDA.

In this section, the band structure and the frequency response of the metamaterial described in the previous section are computed. In theory, the 2D lattice presented in Figure 7 works as a passband or stopband filter, where for the infinite case without damping, perfect filtering properties occur. The parameter values are prescribed such that bandgaps can be obtained in the lower to intermediate frequency range, namely between 100 and 200 (Hz). Specifically, the masses ($m_{L}{,\;m}_{D}$), springs ($k_{x}=m_{L}{\left({2\pi f}_{x}\right)}^{2}{,\;k}_{y}=m_{L}{\left({2\pi f}_{y}\right)}^{2}$) and the damping ratios ($\zeta_{x}{,\;\zeta }_{y}$) in the model were chosen as or the case where damping is considered, according to Table 1.

In the first case, no amplification mechanism is considered. This baseline configuration is then compared with the model that incorporates the DDA connected to the masses $m_{D}$. Then the effect of the number of unit cells along each direction is investigated, followed by the effect of the amplification angle (ϕ) in the dynamic damping properties of the lattice. Finally, the location of the response point on the lattice is briefly examined based on the frequency response functions in characteristic nodes of the structure.

### 3.1. Dynamic Amplification Induced Bandgaps

**Figure 8:**
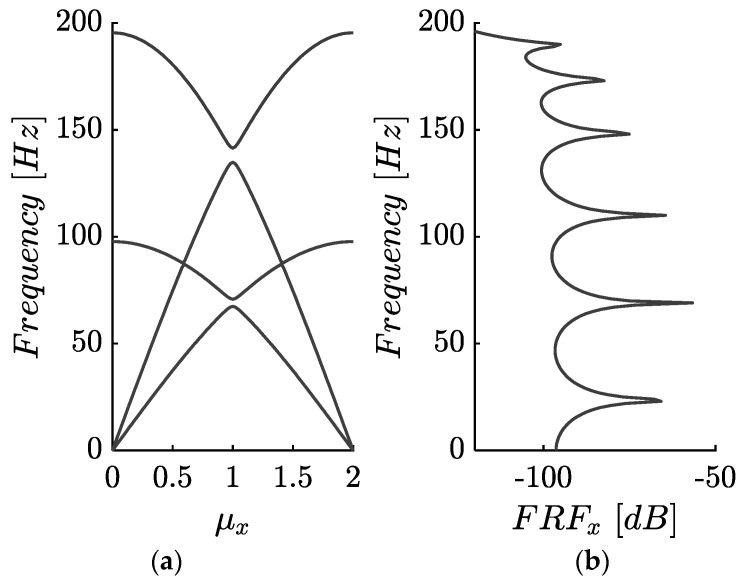
(**a**) Dispersion curves and (**b**) frequency response of the 2D phononic lattice without the DDA along the Γ-X.

Figure 7a corresponds to the considered lattice without the DDA mechanisms and excluding any damping elements. Figure 8 shows the resulting dispersion curves and the corresponding frequency response (FRF) of the 8 × 8 finite periodic lattices at point A along the x-axis. As expected, there are four branches and a Bragg gap between them. The observed bandgap is generated between 135 and 141 (Hz) and is small as the two masses $m_{L}$ and $m_{D}$, have been selected in such a way that there is only a 10% difference between them. Naturally, this small band is hardly visible at the ${FRF}_{x}$.

**Figure 9:**
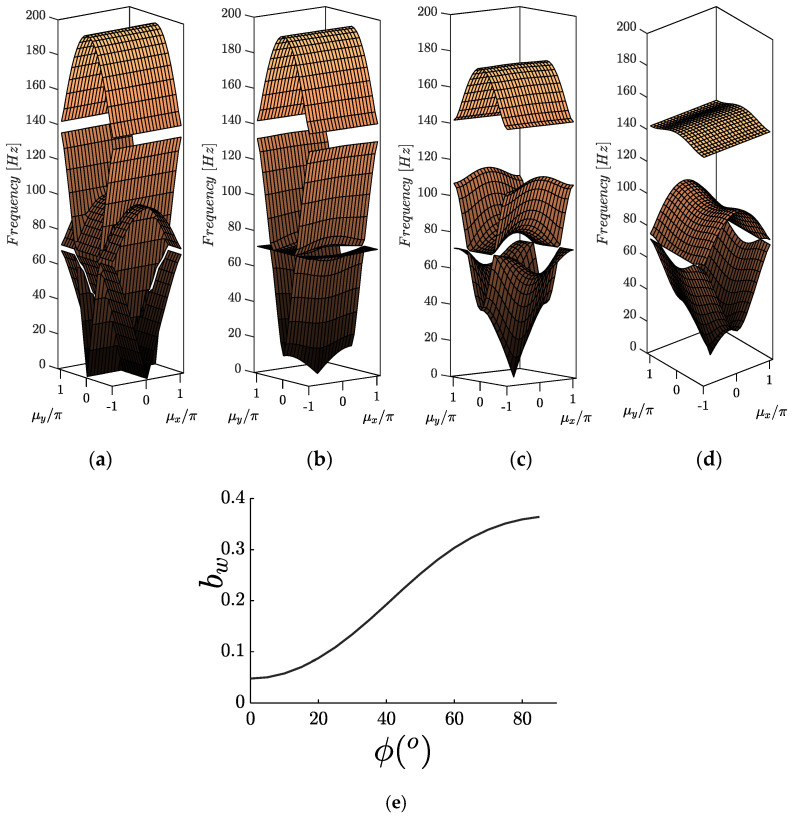
Dispersion contours of 2D phononic lattice for $m_{L}=1.0$, $m_{D}=1.1$, $f_{x\;}=100$ and $f_{y\;}=50$ for the (**a**) lattice without DDA, (**b**) the lattice with DDA and $\phi {(}^{{}^{\circ}})=15$, (**c**) the lattice with DDA and $\phi {(}^{{}^{\circ}})=45$, (**d**) the lattice with DDA and $\phi {(}^{{}^{\circ}})=75$. (**e**) Normalized bandgap width as a function of the amplifier’s angle.

Figure 7b corresponds to the considered lattice, including DDA mechanisms. To show the characteristics of amplification induced phononic gaps in infinite periodic structures, $\phi {(}^{{}^{\circ}})$ is varied from 15 to 75. The band structure of the infinite lattice is obtained through solving Equation (18) for ω by evaluating $\mu_{x}$ and $\mu_{y}$. Figure 9a–d illustrates the “phase constant” surfaces in 3D, where the z-axis shows the frequency corresponding to each pair of normalized wavenumbers ($\mu_{x}{,\;\mu }_{y})$. Table 2 presents the lower and upper limits for the gaps and the normalized bandwidth for the studied cases, which is defined as:

(24) $$b_{w}{=(f}_{u}-{\;f}_{l}{)/f}_{av}{\;,\;f}_{av}{=(f}_{u}{+f}_{l})/2$$

where $f_{Ll}$ and $f_{u}$ correspond to the lower and upper bandgap limits, respectively, and $f_{av}$ is the mid-gap frequency. The normalized bandwidths are also plotted against angle ϕ in Figure 9e.

Comparing the dispersion surfaces of the baseline structure with the one, including the DDAs, reveals that the introduction of amplifiers to the lattice plays a critical role in the size of the forming bandgaps. In addition, the number of dispersion surfaces is reduced to three due to coupling between the vertical (v) and horizontal (u) DoFs of the amplified mass, revealing a second small partial bandgap, which is not of interest in the current study. The amplification effect of mass $m_{D}$ is translated to a gradual “flattening” of the intermediate iso-surface, increasing the distance between the two surfaces. For example, increasing the amplifiers angle $\phi {(}^{{}^{\circ}})$ from 15 to 75 leads to an approximate 600% increase in the bandgap width, clearly showing the beneficial effect of the DDA on the phononic structure.

### 3.2. Effect of Number of Unit Cells

**Figure 10:**
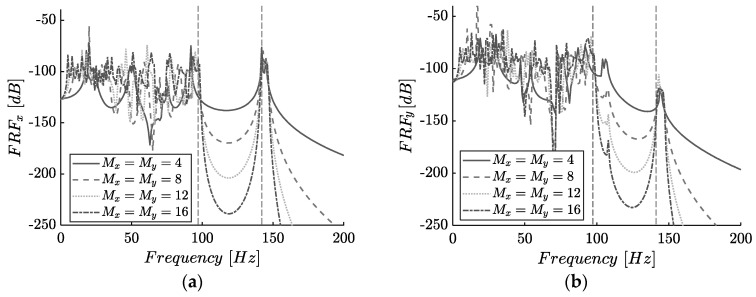
Frequency response (FRF) plot in point A (according to Figure 7a of the $M_{x}{\times M}_{y}$ finite lattice, without damping and amplifier angle $\phi {(}^{{}^{\circ}})=75$ for (**a**) x-direction (**b**) y-direction.

In general, the attenuation of the signal in the bandgap frequency range is lower when only a few units are utilized. Here, for the sake of comparison, we retain the total mass and stiffness of the studied undamped lattices while increasing the number of unit cells. The repetition of unit cells, as presented in Figure 10 for $M_{x}{=M}_{y}=4,\;8,\;12,\;16$ unit -cells, increases the depth of the bandgap while bearing a marginal effect on the high-frequency behavior of the system. Figure 10a displays the FRF in the x-direction of the last mass in the lattice (point A) when subjected to a periodic loading according to Figure 7a. Additionally, the corresponding FRF in the y-direction is depicted in Figure 7b to display the coupling effect between the two degrees of freedom. In both cases, the response curves are presented for the parameters tabulated in Table 1, and for comparison, the bandgap boundaries calculated for the infinite lattice are shown with vertical dashed lines. Naturally, the generated bandgaps are more obvious in ${\mathrm{FFR}}_{x}$ where the DDA is fully activated. Nonetheless, even in the transverse direction, a significant amount of filtering occurs. For $M_{x}{=M}_{y}=4$ the bandgap is identifiable from the curve in between 99.2 and 141.4 (Hz), yet the attenuation in response is only marginally larger than the drops being observed among the other resonance frequencies. With more unit cells, the metamaterial offers an over 50 (dB) bandgap depth. Thus, for further examination regarding the behavior of the structure, the number of unit cells is selected as $M_{x}{=M}_{y}=8$.

### 3.3. Dynamically Induced Metadamping

**Figure 11:**
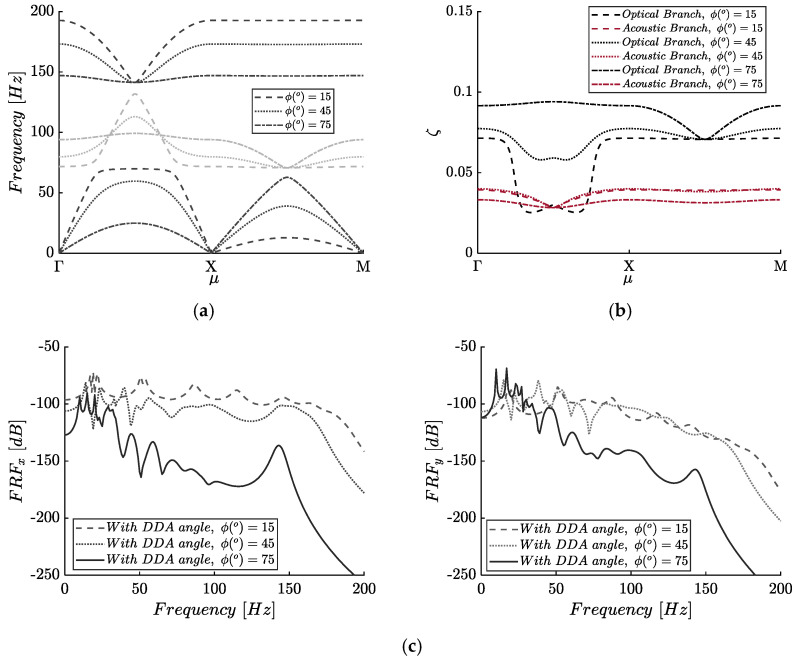
(**a**) Frequency band structure and (**b**) damping ratio (ζ) band structure. (**c**) Frequency response function (FRF) plots of the 8 × 8 finite lattice (response in point A).

Next, the frequency and damping ratio dispersion curves are examined together with the corresponding frequency response functions of the metamaterial for three (3) different values of the amplifier’s angle, as shown in Figure 11. These describe the usual frequency dispersion curves, which now are affected by the presence of damping, i.e., a complex frequency is assumed, together with curves associated with the attenuation of each Bloch mode. Concerning the damping prescription, we assume dashpots connecting the masses on the horizontal and vertical directions with damping ratios $\zeta_{x}=0.02$ and $\zeta_{y}=0.05$. The aim of this investigation is to show the emergent metadamping phenomenon [43] accruing from the dynamic directional amplifier. In other words, the prescribed damping ratio remains constant while different angles of the DDA are examined. The low prescribed damping ratio value of the considered viscous dampers in the x-direction is justified by the extraordinary amplification that the following figures are indicating. The results show that there are shifts in the frequency band diagrams along the Γ–X due to the presence of the dynamic damping, which can be observed comparing the two extreme cases that are plotted. This behavior is more obvious in the damping ratio dispersion curves. Despite the same initial viscous damping values, in all three cases, the case with $\phi {(}^{{}^{\circ}})=75$ exhibits higher dissipation for both the acoustic and optical branches. Specifically, an increased damping ratio is observed in the optical branch (almost five times higher than the one initially prescribed), while for the lower angle $\phi {(}^{{}^{\circ}})=15,$ the optical branch drops abruptly. At the same time, the acoustic branch experiences little change, which leads to a reduced bandgap. The two branches meet in the low damping ratio region, and the so-called branch-overtaking phenomenon [47] occurs. As expected, this phenomenon does not occur for larger amplification angles. This is an indication of the emergence of dissipation, which is present along the whole spectrum and not only for modes close to the bandgap, as seen in Figure 11c, where the FRF of the damped 8 × 8 finite lattice is plotted. Consequently, the DDA mechanism not only increases the expected bandwidth size but also provides a great example of metadamping emergence.

### 3.4. Effects of Response Point

**Figure 12:**
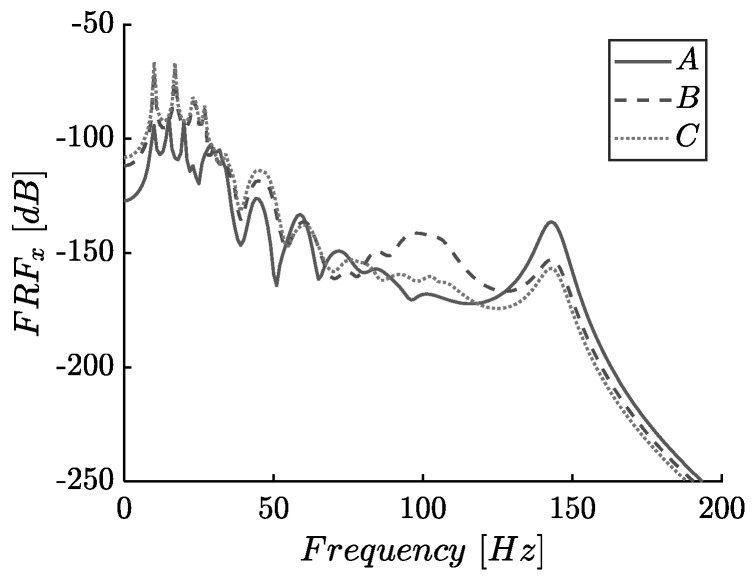
Frequency response (${FRF}_{x}$) of the 8×8 finite lattice, for $\zeta_{0x}=0.02$, $\zeta_{0y}=0.05$, and amplifier’s angle $\phi {(}^{{}^{\circ}})=75$ at points A–C.

A distributed source along the edge nodes excites the longitudinal and transverse modes of Figure 7a finite lattice. As already mentioned, the DDA mechanism is designed to function in the x-direction; hence a different response is expected along the x and y-axes. To select the input and output nodes on this lattice, we remove the fixed boundary conditions on the output points A–C. Figure 12 shows the FRF plots of the 8 × 8 finite periodic lattice for longitudinal excitation along the x-axis, assuming damping ratios $\zeta_{x}=0.02$, $\zeta_{y}=0.05$ and amplifier angle $\phi {(}^{{}^{\circ}})=75$.

Notice that in Figure 12, there are small resonance peaks in the response of output point A at approximately f=50 (Hz) close to the antiresonance notches of output points B, C. These peaks are due to the applied boundary conditions. Nevertheless, the peaks do not affect the gap performances, as they are close to the lower limit of the bandgap. Moreover, at low frequencies (below 25 (Hz)), the FRF of A is shifted downwards due to the rigid body translation modes, while a larger bandgap depth is observed in A compared with the one of the interim point B. The different response observed between point A and C is justified by the existence of an amplified or not mass in the selected node. Except for these differences, the FRF of the presented output points does not show great deviations, revealing that the proposed metamaterial is effective throughout its length.

## 4. Conceptual Design

**Figure 13:**
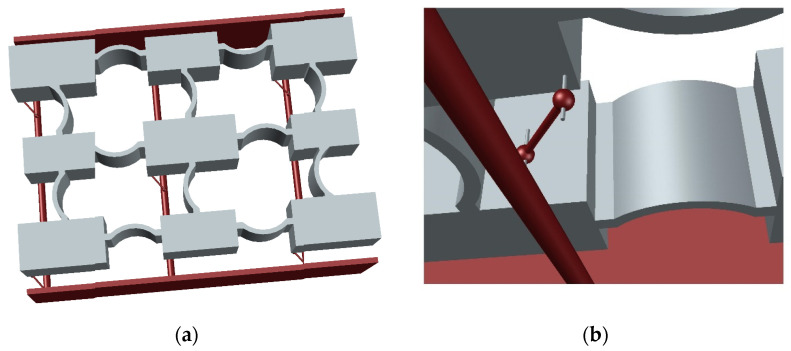
Conceptual design of the proposed metastructure, (**a**) 3D view (**b**) detail of the dynamic directional amplifier (DDA).

Figure 13 presents a conceptual design of the proposed metastructure comprising of a single unit cell (for simplicity), yet larger lattices can be easily constructed by the spatial repetition of this unit cell. The mass–spring lattice is envisaged as a compliant 3D-printed part, supported by the red-colored metal frame. The role of this frame is to accommodate the DDA mechanisms at the specified locations providing overhangs for the pinned connections. The proposed realization can be designed either as a microstructure or on a larger scale, allowing the implementation in various applications, including acoustic and vibration control and seismic isolation.

## 5. Conclusions

The present study demonstrated the theoretical framework of a novel 2D phononic metamaterial consisting of dynamically amplified masses. The novelty lies in the simplicity of the proposed system. The dynamic directional amplification (DDA) mechanism is realized without additional masses or complex geometries since the amplification can be achieved by coupling the kinematic DoFs of the mass with a rigid link. Initially, the effect of coupling between the vertical and horizontal motion of the mass–spring system was shown based on a monoatomic configuration. Then, the enhanced phononic structure was formulated based on the proposed amplification mechanism, and a simple indicative example was demonstrated to show its capabilities. The dispersion relationships illustrated that the amplification of the mass could provide large bandgaps in the low-frequency regime without the need for large parasitic mass addition. We also demonstrated that using dynamic amplification, deep gaps at low frequencies can be obtained in the direction of wave propagation, using a moderate number of unit cells, while an emergent metadamping phenomenon occurs even when minuscule damping ratios values are prescribed. To conclude, the provided indicative implementation of this concept shows great promise towards developing acoustic metamaterial designs able to offer low-frequency isolation. Future research will focus on numerical and experimental validation of the proposed metamaterial based on designs suitable for low-cost production.

## Figures and Tables

**Table 1:** Model parameters.

$m_{L}\;(\mathrm{kg})$	$m_{D}\;(\mathrm{kg})$	$f_{0x}\;(\mathrm{Hz})$	$f_{0y}\;(\mathrm{Hz})$	$\zeta_{x}$	$\zeta_{y}$
1.0	1.1	100	50	0.02	0.05

**Table 2:** Lower ($f_{l})$, upper ${(f}_{u})$ bandgap limits and normalized gap widths ${(b}_{w})$.

Case	$f_{u}\;(\mathrm{Hz})$	$f_{l}\;(\mathrm{Hz})$	$f_{\mathrm{av}}\;(\mathrm{Hz})$	$b_{w}$
Without DDA	141.4	134.8	138.1	0.05
$\phi {(}^{{}^{\circ}})=15$	141.4	131.9	136.6	0.07
$\phi {(}^{{}^{\circ}})=45$	141.4	113.2	127.3	0.22
$\phi {(}^{{}^{\circ}})=75$	141.4	99.2	120.3	0.35

## Data Availability

Data Sharing is not applicable.

## Appendix A

Following is the derivation of Equation (10). In Figure 3, connecting the amplification mass to the origin of the local CSYS via a hinged, rigid, massless rod imposes a kinematic constraint between the degrees of freedom u and v. The lumped parameter model is described by the coordinates of the amplification mass (m) in a random position ${A(x,y)=(x}_{0}{+u,y}_{0}-v)$, the initial angle between the horizontal axis and the link $\varphi^{{}^{\circ}}=\mathrm{atan}(\frac{x_{0}}{y_{0}})$ and the rotation of the rod $\theta^{{}^{\circ}}$ at the random position A of the moving mass. Then the kinetic (T) and potential (V) energies of the system are calculated as:

(A1) $$T=\frac{1}{2}m\left({\dot{u}}^{2}{+\dot{v}}^{2}\right)=\frac{1}{2}m\left(1+\frac{x^{2}}{y^{2}}\right){\dot{u}}^{2}=\frac{1}{2}{mL}^{2}\frac{{\dot{u}}^{2}}{y^{2}}\;$$

(A2) $$V=\frac{1}{2}k_{x}{\left(l_{x}-l_{xi}\right)}^{2}+\frac{1}{2}k_{x}{\left(l_{y}-l_{yi}\right)}^{2}$$

where $l_{xi}$, $l_{yi}$ and $l_{x}$, $l_{y}$ are the deformations of the springs at the initial and a random position, respectively.

For L = T–U and by application of the Lagrange principle:

(A3) $$\frac{d}{\mathrm{dt}}\;(\frac{\partial T}{\partial \dot{u}})\;-\frac{\partial T}{\partial u}+\frac{\partial V}{\partial u}=0\;$$

the equation of motion is obtained after linearization as:

(A4) $$\frac{{mL}^{2}}{y_{0}^{2}}\ddot{u}+\left[k_{x}{+k}_{y}{\left(\frac{x_{0}}{y_{0}}\right)}^{2}\right]u=F$$

which can be rewritten as:

(A5) $$m\left[{1+\tan}^{2}\left(\varphi \right)\right]\ddot{u}+\left[k_{x}{+k}_{y}{\;\tan}^{2}\left(\varphi \right)\right]u=F$$

or for ρ = tanφ

(A6) $$m\left[{1+\rho }^{2}\right]\ddot{u}+\left[k_{x}{+k}_{y}\rho^{2}\right]u=F$$

## Appendix B

In this appendix, we provide the explicit form of the **M**, **C**, **K** matrices used in the dispersion equation of the lattice without the DDA–Equation (17) and, including the DDA—Equation (18). Starting with the lattice without the amplification mechanism, the dispersion relationship can be calculated from:

(A7) $${\det\;[M}_{p}\ddot{u}+C_{p}\dot{u}+K_{p}u]=0$$

with the $M_{p}$, $C_{p}$ and $K_{p}$ matrices defined as follows:

(A8) $$M_{p}=\left[\begin{array}{cccc} m_{L} & 0 & 0 & 0\\ 0 & m_{L} & 0 & 0\\ 0 & 0 & m_{D} & 0\\ 0 & 0 & 0 & m_{D} \end{array}\right],$$

(A9) $$C_{p}=\left[\begin{array}{cccc} {2c}_{x} & 0 & -c_{x}(1+e^{{-i\mu }_{x}}) & 0\\ 0 & {2c}_{y} & 0 & -c_{y}e^{-\frac{{i\mu }_{x}}{2}}\left(e^{-\frac{{i\mu }_{y}}{2}}+e^{\frac{{i\mu }_{y}}{2}}\right)\\ -c_{x}{(1+e}^{{i\mu }_{x}}) & 0 & {2c}_{x} & 0\\ 0 & -c_{y}e^{\frac{{i\mu }_{x}}{2}}\left(e^{-\frac{{i\mu }_{y}}{2}}+e^{\frac{{i\mu }_{y}}{2}}\right) & 0 & {2c}_{y} \end{array}\right]$$

(A10) $$K_{p}=\left[\begin{array}{cccc} {2k}_{x} & 0 & -k_{x}{(1+e}^{-{\;i\mu }_{x}}) & 0\\ 0 & {2k}_{y} & 0 & -k_{y}e^{-\frac{{i\mu }_{x}}{2}}\left(e^{-\frac{{i\mu }_{y}}{2}}+e^{\frac{{i\mu }_{y}}{2}}\right)\\ -{\;k}_{x}{(1+e}^{{i\mu }_{x}}) & 0 & {2k}_{x} & 0\\ 0 & -{\;k}_{y}e^{\frac{{i\mu }_{x}}{2}}\left(e^{-\frac{{i\mu }_{y}}{2}}+e^{\frac{{i\mu }_{y}}{2}}\right) & 0 & {2k}_{y} \end{array}\right]$$

Finally, the system with the amplified masses $m_{D}$ can be calculated by multiplying $M_{p}{,\;C}_{p}$ and $K_{p}$ by the transformation matrix:

(A11) $$Q_{p}=\left[\begin{array}{ccc} 1 & 0 & 0\\ 0 & 1 & 0\\ 0 & 0 & 1\\ 0 & 0 & \rho \end{array}\right]$$

The dispersion relationship of the phononic structure with directional inertial amplifiers is given by:

(A12) $$\det\left[-s^{2}M_{p,a}+{sC}_{p,a}+K_{p,a}\right]=0$$

where

(A13) $$M_{p,a}{=Q}_{p}^{T}M_{p}Q_{p}=\left[\begin{array}{ccc} m_{L} & 0 & 0\\ 0 & m_{L} & 0\\ 0 & 0 & m_{D}{(1+\rho }^{2}) \end{array}\right]$$

(A14) $$C_{p,\alpha }{=Q}_{p}^{T}C_{p}Q_{p}=\left[\begin{array}{ccc} 2c_{x} & 0 & -c_{x}{(1+e}^{-{\;i\mu }_{x}})\;)\\ 0 & {2c}_{y} & {2\rho c}_{y}e^{\frac{-{\;i\mu }_{x}}{2}}\cos\left(\frac{\mu_{y}}{2}\right)\\ -{\;c}_{x}{(1+e}^{{i\mu }_{x}}) & {2\rho c}_{y}e^{\frac{{i\mu }_{x}}{2}}\cos\left(\frac{\mu_{y}}{2}\right) & {2c}_{x}+{2\rho }^{2}c_{y} \end{array}\right]$$

(A15) $$K_{p,\alpha }{=Q}_{p}^{T}K_{p}Q_{p}=\left[\begin{array}{ccc} {2k}_{x} & 0 & -k_{x}{(1+e}^{-{i\mu }_{x}})\;)\\ 0 & {2k}_{y} & {2\rho k}_{y}e^{\frac{-{\;i\mu }_{x}}{2}}\cos\left(\frac{\mu_{y}}{2}\right)\\ -k_{x}{(1+e}^{{i\mu }_{x}}) & {2\rho k}_{y}e^{\frac{{i\mu }_{x}}{2}}\cos\left(\frac{\mu_{y}}{2}\right) & {2k}_{x}+{2\rho }^{2}k_{y} \end{array}\right]$$

## Appendix C

In this appendix, we provide the explicit form of Equation (20) of the lattice with and without the DDA for $M_{x\;}{=M}_{y}=2$.

The equation of motion of the meta-material without the DDA, for 2 × 2 number of units is expressed in matrix formulation as:

(A16) $$M\ddot{u}(t)+C\dot{u}{(t)+\mathrm{Ku}(t)=Fe}^{st}$$

where $M_{8\times 8}$, $C_{8\times 8}$, $K_{8\times 8}$,$\;{\ddot{u}}_{8\times 1}$, ${\dot{u}}_{8\times 1}$ $u_{8\times 1}$, $F_{8\times 1}$. The Global mass ${[M}_{p}^{G}]$, damping ${[C}_{p}^{G}]$ and stiffness ${[K}_{p}^{G}]$ matrices are defined as follows:

(A17) $$M_{p}^{G}=\left[\begin{array}{cccccccc} m_{L} & 0 & 0 & 0 & 0 & 0 & 0 & 0\\ 0 & m_{L} & 0 & 0 & 0 & 0 & 0 & 0\\ 0 & 0 & m_{D} & 0 & 0 & 0 & 0 & 0\\ 0 & 0 & 0 & m_{D} & 0 & 0 & 0 & 0\\ 0 & 0 & 0 & 0 & m_{D} & 0 & 0 & 0\\ 0 & 0 & 0 & 0 & 0 & m_{D} & 0 & 0\\ 0 & 0 & 0 & 0 & 0 & 0 & m_{L} & 0\\ 0 & 0 & 0 & 0 & 0 & 0 & 0 & m_{L} \end{array}\right]$$

(A18) $$C_{p}^{G}=\left[\begin{array}{cccccccc} {2c}_{x} & 0 & -c_{x} & 0 & 0 & 0 & 0 & 0\\ 0 & {2c}_{y} & 0 & 0 & 0 & -c_{y} & 0 & 0\\ -c_{x} & 0 & c_{x} & 0 & 0 & 0 & 0 & 0\\ 0 & 0 & 0 & {2c}_{y} & 0 & 0 & 0 & -c_{y}\\ 0 & 0 & 0 & 0 & {2c}_{x} & 0 & -c_{x} & 0\\ 0 & -c_{y} & 0 & 0 & 0 & c_{y} & 0 & 0\\ 0 & 0 & 0 & 0 & -{\;c}_{x} & 0 & c_{x} & 0\\ 0 & 0 & 0 & -{\;c}_{y} & 0 & 0 & 0 & c_{y} \end{array}\right],$$

(A19) $$K_{p}^{G}=\left[\begin{array}{cccccccc} {2k}_{x} & 0 & -{\;k}_{x} & 0 & 0 & 0 & 0 & 0\\ 0 & {2k}_{y} & 0 & 0 & 0 & -{\;k}_{y} & 0 & 0\\ -k_{x} & 0 & k_{x} & 0 & 0 & 0 & 0 & 0\\ 0 & 0 & 0 & {2k}_{y} & 0 & 0 & 0 & -k_{y}\\ 0 & 0 & 0 & 0 & {2k}_{x} & 0 & -{\;k}_{x} & 0\\ 0 & -{\;k}_{y} & 0 & 0 & 0 & k_{y} & 0 & 0\\ 0 & 0 & 0 & 0 & -k_{x} & 0 & k_{x} & 0\\ 0 & 0 & 0 & -{\;k}_{y} & 0 & 0 & 0 & k_{y} \end{array}\right]$$

Similarly, for the lattice with the DDA, for $M_{x}{=M}_{y}=2$ the global mass ${[M}_{p,\alpha }^{G}]$, damping ${[C}_{p,\alpha }^{G}]$ and stiffness ${[K}_{p,\alpha }^{G}]$ matrices can be calculated from:

(A20) $$M_{p,\alpha }^{G}{=Q}_{G}^{T}M_{p}^{G}Q_{G},$$

(A21) $$C_{p,\alpha }^{G}{=Q}_{G}^{T}C_{p}^{G}Q_{G}$$

(A22) $$K_{p,\alpha }^{G}{=Q}_{G}^{T}K_{p}^{G}Q_{G}$$

where the transform matrix in this case is:

(A23) $$Q^{G}=\left[\begin{array}{cccccc} 1 & 0 & 0 & 0 & 0 & 0\\ 0 & 1 & 0 & 0 & 0 & 0\\ 0 & 0 & 1 & 0 & 0 & 0\\ 0 & 0 & \rho & 0 & 0 & 0\\ 0 & 0 & 0 & 1 & 0 & 0\\ 0 & 0 & 0 & \rho & 0 & 0\\ 0 & 0 & 0 & 0 & 1 & 0\\ 0 & 0 & 0 & 0 & 0 & 1 \end{array}\right],$$
